# Efficacy and safety of *N*-acetyl-l-leucine in Niemann–Pick disease type C

**DOI:** 10.1007/s00415-021-10717-0

**Published:** 2021-08-13

**Authors:** Tatiana Bremova-Ertl, Jens Claassen, Tomas Foltan, Jordi Gascon-Bayarri, Paul Gissen, Andreas Hahn, Anhar Hassan, Anita Hennig, Simon A. Jones, Miriam Kolnikova, Kyriakos Martakis, Jan Raethjen, Uma Ramaswami, Reena Sharma, Susanne A. Schneider

**Affiliations:** 1 Department of Neurology, University Hospital Bern (Inselspital), 3010 Bern, Switzerland; 2 Department of Neurology, University Hospital Essen, University of Duisburg-Essen, Essen, Germany; 3 Department of Neurocritical Care, Neurological and Neurosurgical First Stage Rehabilitation and Weaning, MediClin Klinik Reichshof, Reichshof-Eckenhagen, Germany; 4 Department of Pediatric Neurology, National Institute of Children’s Diseases, Comenius University in Bratislva, Bratislva, Slovak Republic; 5 Department of Neurology, Bellvitge University Hospital, L’Hospitalet de Llobregat, Spain; 6 NIHR Great Ormond Street Hospital Biomedical Research Centre, University College London, London, UK; 7 Department of Child Neurology, Justus Liebig University, Giessen, Germany; 8 Department of Neurology, Mayo Clinic, Rochester, MN USA; 9 Department of Neurology, Ludwig Maximilians University, Munich, Germany; 10 Willink Unit, Manchester Centre for Genomic Medicine, Royal Manchester Children’s Hospital, University of Manchester, Manchester, UK; 11 Neurology Outpatient Clinic, Kiel, Germany; 12 Medical Faculty, Christian Albrechts University Kiel, Kiel, Germany; 13 Lysosomal Storage Disorder Unit, Royal Free London NHS Foundation Trust, London, UK; 14 Department of Adult Metabolic Medicine, Salford Royal Foundation NHS Trust, Salford, UK

**Keywords:** Niemann–Pick disease type C, Lysosomal storage disorder, Ataxia, Acetyl-leucine, Symptomatic therapy

## Abstract

**Objective:**

To investigate the safety and efficacy of *N*-acetyl-l-leucine (NALL) on symptoms, functioning, and quality of life in pediatric (≥ 6 years) and adult Niemann–Pick disease type C (NPC) patients.

**Methods:**

In this multi-national, open-label, rater-blinded Phase II study, patients were assessed during a baseline period, a 6-week treatment period (orally administered NALL 4 g/day in patients ≥ 13 years, weight-tiered doses for patients 6–12 years), and a 6-week post-treatment washout period. The primary Clinical Impression of Change in Severity (CI-CS) endpoint (based on a 7-point Likert scale) was assessed by blinded, centralized raters who compared randomized video pairs of each patient performing a pre-defined primary anchor test (8-Meter Walk Test or 9-Hole Peg Test) during each study periods. Secondary outcomes included cerebellar functional rating scales, clinical global impression, and quality of life assessments.

**Results:**

33 subjects aged 7–64 years with a confirmed diagnosis of NPC were enrolled. 32 patients were included in the primary modified intention-to-treat analysis. NALL met the CI-CS primary endpoint (mean difference 0.86, SD = 2.52, 90% CI 0.25, 1.75, *p* = 0.029), as well as secondary endpoints. No treatment-related serious adverse events occurred.

**Conclusions:**

NALL demonstrated a statistically significant and clinical meaningfully improvement in symptoms, functioning, and quality of life in 6 weeks, the clinical effect of which was lost after the 6-week washout period. NALL was safe and well-tolerated, informing a favorable benefit-risk profile for the treatment of NPC.

**Clinicaltrials.gov identifier:**

NCT03759639.

**Supplementary Information:**

The online version contains supplementary material available at 10.1007/s00415-021-10717-0.

## Introduction

Niemann–Pick disease type C (NPC) is a rare (incidence 1:100,000), prematurely fatal, autosomal recessive, neurovisceral lysosomal storage disease that predominantly affects children. However, adolescent and adult-onset cases are being increasingly recognized [1]. The disease typically begins in early childhood, is chronic, progressive, and severely reduces the quality of life. The presentation of NPC is characterized by broad heterogeneity in systemic, psychiatric, and neurological symptoms, which depend on the age of onset of neurological symptoms [2]. There is broad inter-individual phenotypic variability, including age at onset and rate of progression. This renders an assembly of well-matched cohorts of NPC patients for controlled trials difficult to achieve.

Treatment of NPC is currently limited to reducing the rate of disease progression with the substrate reduction therapy Miglustat (Zavesca™), approved in the European Union and several other countries, but not in the United States.

*N*-Acetyl-l-leucine (NALL) is the l-enantiomer of *N*-acetyl-dl-leucine, a modified, acetylated derivative of a natural essential amino acid (Leucine) that has been available in France since 1957 as a racemate (equal amounts of both d- and l-enantiomers) under the trade name Tanganil™ (Pierre Fabre Laboratories, France) as a treatment for acute vertigo. Prior observational studies assessing the effect of *N*-acetyl-dl-leucine (racemic mixture) in patients with NPC suggest a beneficial symptomatic, and neuroprotective, disease-modifying effect. In a case series, short-term treatment with *N*-acetyl-dl-leucine was found to improve ataxia, cognition, and quality of life in 12 patients with NPC. Subsequent long-term case series and pre-clinical studies demonstrated the neuroprotective, disease-modifying effect of treatment in NPC [3–5]. In all, the compound was well tolerated with no serious side effects.

Animal studies in the NPC mouse model (*Npc1*^*−/−*^) have shown the l-enantiomer, i.e. NALL, has clinical benefits over the racemate. Treatment with *N*-acetyl-dl-leucine and its enantiomers all significantly reduced ataxia when commenced pre-symptomatically (from 3-weeks of age onward) and symptomatically (for 1-week treatment, starting at 8 weeks of age). However, only NALL significantly delayed the onset of functional decline (gait abnormalities, motor dysfunction), the decline in general health and condition, slowed disease progression, and prolonged survival—whereas the d-enantiomer did not). One mechanism of action of NALL that has been implicated for this neuroprotective, disease-modifying effect is the activation of cerebral glucose metabolism in the cerebellum, correlated with enhanced cerebellar activity [5]. Pharmacokinetic studies also suggest the d-enantiomer could accumulate relative to the l-enantiomer during chronic administration of the racemate, with the potential for long-term negative effects [6]. Therefore, the individual l-enantiomer is being developed.

## Methods

### Study design

The IB1001-201 clinical trial was one of three multi-national, open-label, rater-blinded trials that utilize a single master protocol to investigate NALL (Sponsor Code IB1001) for the treatment of three rare, neurodegenerative diseases [in addition to NPC, GM2 Gangliosidosis (NCT03759678) and Ataxia-Telangiectasia (NCT03759678)]. This IB1001 master protocol was designed through a collaboration between National Regulatory Agencies, leading clinical experts, patient organizations, and the industry sponsor to address the unique ethical and practical challenges to conducting clinical trials for these orphan, heterogeneous patient populations. Details of the trial methods, rationale, design, and oversight have been previously published [7].

The IB1001-201 clinical trial was separated into two study phases to enable the investigation of both the symptomatic (“Parent Study”), and long-term (“Extension Phase”) safety and efficacy of NALL. The results of the Parent Study are reported below. The Extension Phase is ongoing.

### Participants

The eligibility criteria have previously been published [7].

### Standard protocol approvals, registrations, and patient consents

Approval was obtained by the applicable responsible central research ethics committees/institutional review boards for each center (Ethics Committee of Ludwig Maximilian University of Munich (19-135), Slovakia: National Institute of Child Diseases Bratislava Ethics Committee (EudraCT 2018-004431-71), Bellvitge Hospital University Clinical Research Ethics Committee (AC001/19), East Midlands—Derby Research Ethics Committee (259038), Mayo Clinic Institutional Review Board (18-011974). Written informed consent was obtained for all study participants by the subject or, if applicable, their parent or legal representative. The study is registered with ClinicalTrials.gov NCT03759639, EudraCT number 2018-004331-71, and DR KS-ID: DRKS00016567.

### Procedures

**Fig. 1:**
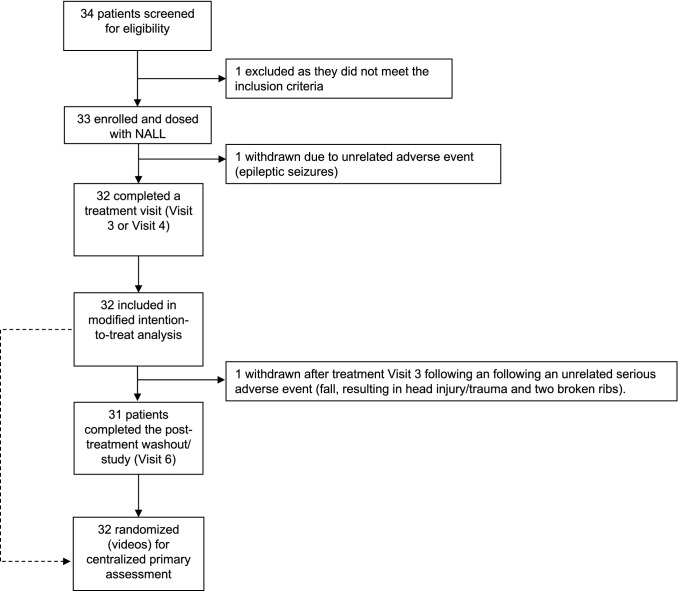
Trial profile

Adult and pediatric subjects with a confirmed diagnosis per the current recommendations for the detection and diagnosis of NPC were [8] recruited at 9 clinical research Universities and Hospitals in five countries (Germany, Slovakia, Spain, the United States, and the United Kingdom) (Fig. 1). The Parent Study consisted of three study periods: a baseline period (with or without a study run-in washout from prohibited medications), a treatment period, and a post-treatment washout period, with two patient visits per period. Duration of the treatment phase and washout phase were 42 days (+ 7 days) each, per protocol. No subject randomization of participants/allocation of participants to different study arms occurred (i.e., to different intervention arms). However, subject videos obtained from each study period were randomized and assessed as the basis of the primary endpoint by blinded, centralized raters. The schedule of events is reported in Table 1. This study was ongoing during the Coronavirus Pandemic (COVID-19), which significantly impacted the schedule of events.

**Table 1:** Schedule of assessments

Period	Baseline period		Treatment period		Wash-out period		Early term
Duration of the whole period	1 day	2 weeks	6 weeks		6 weeks		1 day
Visit number	Visit 1	Visit 2	Visit 3	Visit 4	Visit 5	Visit 6/EOS	ET
Name of the visit	Screening/Bsl 1	Baseline 2	Treatment 1	Treatment 2	Washout 1	Washout 2	ET
Timeline (days)	Day-14	Day 1, Start IMP	Day 28	Day 42	Day 70	Day 84	XX
Visit window allowed	na	+ 7 days	+ 7 days	+ 7 days	+ 7 days	+ 7 days	na

Patient information and informed consent process	X						
Inclusion/exclusion criteria, medical history, patient demographics	X	X					
Classify patient as “Naïve” or “Non-naïve”	X						
Documentation of therapy and concomitant medications	X	X	X	X	X	X	X
Vital signs	X	X	X	X	X	X	X
12-lead electrocardiogram (ECG)	X		X		X		X
Urine test for *N*-Acetyl-d-Leucine	X	X			X	X	X
Blood safety laboratory tests and urinalysis	X	X	X	X	X	X	X
Blood sample for sparse PK	X	X	X	X	X	X	X
Quality of Life EQ-5D	X	X	X	X	X	X	X
Scale for Ataxia Rating (SARA)	X	X	X	X	X	X	X
Modified Disabling Rating Score (mDRS)	X	X	X	X	X	X	X
Scale for Spinocerebellar Ataxia Functional Index (SCAFI)	X	X	X	X	X	X	X
CI-CS Anchor Test Video Record	X	X	X	X	X	X	X
Clinical Global Impression of Severity (CGI-S)	X	X	X	X	X	X	X
Clinical Global Impression of Change (CGI-C) by Physician, Caregiver, Patient				X		X	X
Documentation of AEs	X	X	X	X	X	X	X

At the initial screening visit, patients were classified as either “naïve” or “non-naïve” depending on their use of prohibited medications (e.g., *N*-acetyl-dl-leucine) within the past 6 weeks. “Non-naïve” patients were given the one-time opportunity to undergo a minimum of 42 days washout before returning for the baseline 1 visit.

During the treatment period, patients aged ≥ 13 years or aged 6–12 years weighing ≥ 35 kg received 4 g/day of orally administered NALL (powder for suspension in 40 mL Ora-Blend^®^) three times per day (2 g in the morning, 1 g in the afternoon, and 1 g in the evening). Patients aged 6–12 years weighing < 35 kg received weight-tiered doses two or three times per day based on approximately 0.1 g/kg/day.

After their final visit of the Parent Study (Visit 6), patients could enter a long-term, open-label extension study which is ongoing.

### Outcomes

The primary endpoint was the novel, functionally relevant Clinical Impression of Change in Severity (CI-CS). The CI-CS assessment was performed by blinded, centralized raters who compared paired videos of each patient performing a “primary anchor test” [either the 8 Meter Walk Test (8MWT) or 9 Hole Peg Test-Dominant Hand (9HPT-D)] at baseline (Visit 2), end of treatment (Visit 4), and end of washout (Visit 6) study visits. Blinded to the time point of each video in each pair, the raters made an objective comparison scored on a 7-point Likert scale of the change in the severity of the patient’s neurological signs and symptoms from Video 1 to Video 2. Details of CI-CS administration and assessment have been previously published [7].

Each patient’s primary anchor test (8MWT or 9HPT-D) was selected by the Principal Investigator at Visit 1 based on the patient’s unique clinical symptoms to better ensure the clinical relevancy of the primary outcome assessment. The anchor tests were filmed in a standardized way at each visit and uploaded for centralized review. A pool of three board-certified neurologists performed the central video analysis. Two “primary raters” were responsible for the initial comparison of the video pairs. For cases where the two initial reviewers differed in their assessment of the primary CI-CS score by more than one (1) point, the third rater acted as an adjudicator.

The raters assessed: “Compared to the first video, how has the severity of the patient’s performance on the 8MWT or 9HPT-D changed (improved or worsened) as observed in the second video?” The CI-CS assessment was based on a 7-point Likert scale ranging from + 3 (significantly improved) to − 3 (significantly worse).

Secondary assessments were analyzed between two time points: (1) baseline (Visit 2) to the end of treatment (Visit 4); (2) the end of treatment (Visit 4) to the end of post-treatment washout (Visit 6).

To evaluate the overall neurologic status in NPC disease, the modified Disability Rating Scale (mDRS) was applied, consisting of 6 subdomains (ambulation, manipulation, Seizures, Language, Swallowing, Ocular movements) [9]. Cerebellar function evaluations were administered, including: (1) the Scale for the Assessment and Rating of Ataxia (SARA), an 8-item clinical rating scale (gait, stance, sitting, speech, fine-motor function, and coordination; range 0–40, where 0 is the best neurologic status and 40 the worst) [10]; and (2) the Spinocerebellar Ataxia Functional Index (SCAFI), comprising 8-m walking time performed by having patients walk twice, as quickly as possible, from one line to another excluding turning, the 9-Hole Peg Test (9HPT) with the dominant and non-dominant hand, and the number of “PATA” repetitions over 10 s (PATA) [11].

Subjective impairment and quality of life were evaluated by using the Clinical Global Impression of Severity and Improvement Scale (completed by the Investigator, Caregiver, and Subject) [12], the EuroQol (EQ) 5Q-5D-5L/Y questionnaire and the visual analog scale (VAS) [13].

Safety assessments included adverse event monitoring, clinical laboratory testing, and sparse pharmacokinetic sampling, and collecting vital signs and electrocardiograms.

### Randomization and masking

CI-CS anchor test videos (8MWT and 9HPT-D) were submitted directly from trial sites to a third-party vendor, Medpace Core Laboratories (MCL). For each patient, MCL generated a random number (1–6) via *RANDOM.ORG* which corresponded to a video analysis order sequence (Supplementary Table 1). Once all applicable videos for a patient had been obtained, MCL assigned the patient’s videos to this randomization sequences, generating 3 randomized video pairs for the CI-CS assessment. These 3 CI-CS assessments were released to the blinded raters for review via the secure MCL Clintrak Imaging System Portal.

Only the MCL IB1001 study team had access to the randomization sequences. To ensure that central raters were blinded to any patient identifiers, each patient video was assigned a “barcode” and “reading number” that identified the video throughout the central review process. Usage of the “barcode” and “reading number” blinded the raters to any information (i.e., subject ID, DOB, visit identifier) that might have introduced bias during the central review.

### Statistical analysis

**Fig. 2:**
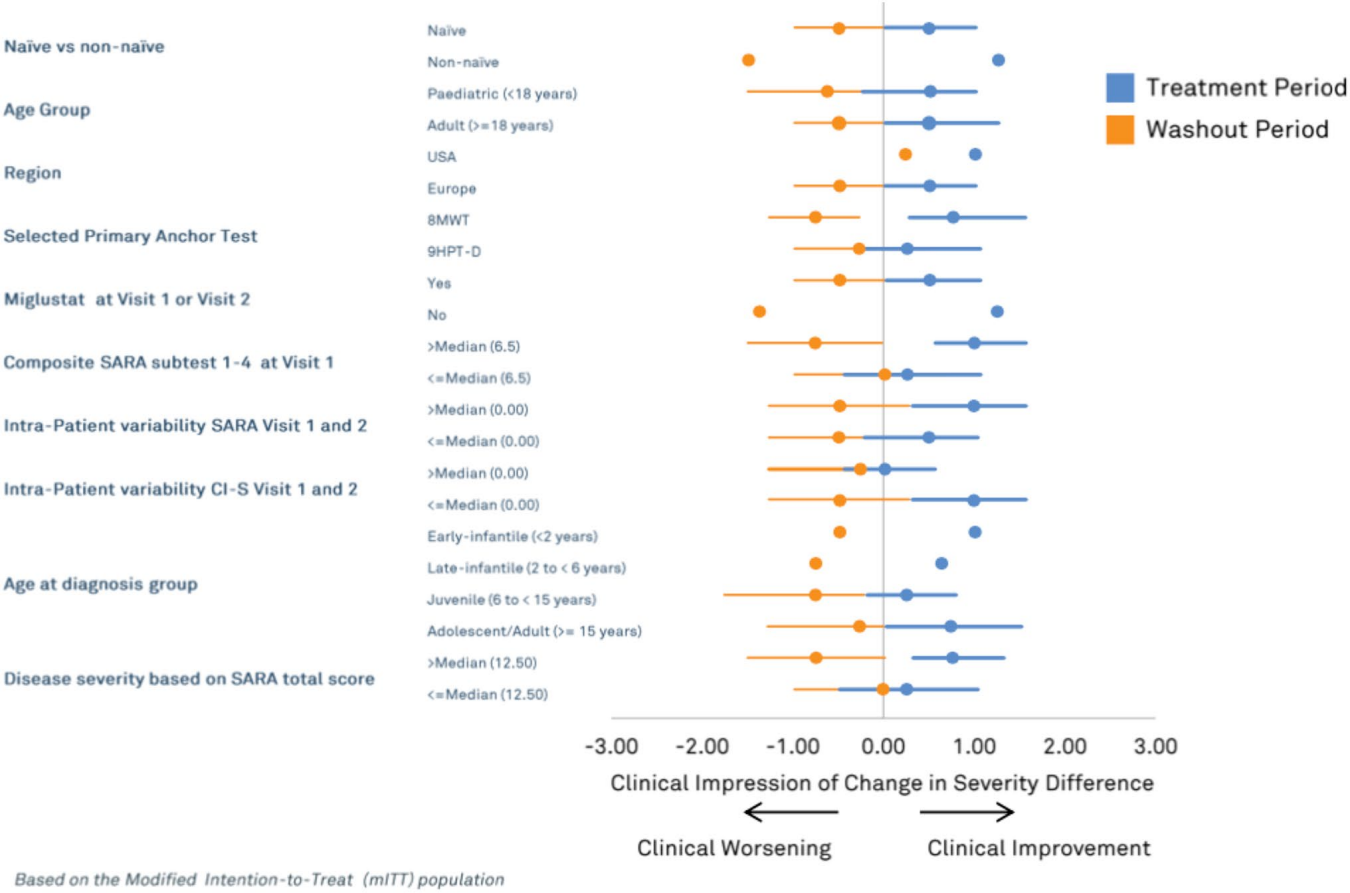
Forest plot for CI-CS scores for pre-defined subgroup analysis, based on the mITT population. The lines and dots in blue represent the change per subgroup on the CI-CS scores during the treatment period: Visit 4 (end of treatment) vs. Visit 2 (baseline). The lines and dots in orange represent the change per subgroup on the CI-CS scores during the washout period: CI-CS scores visit 6 (end of washout) vs. Visit 4 (end of treatment). The dots represent the pseudo-medians or Hodges–Lehmann estimators, the horizontal lines represent the 90% confidence intervals. *LCL* lower confidence limit, *UCL* upper confidence limit. For some subgroups the number of patients was too small to calculate the LCL and/or UCL, in that case, the result is presented as missing and no line presenting the confidence interval is drawn. No last observation carried forward (LOCF) approach was used for this figure. Only values from patients with reported data are included.

The primary endpoint was defined as the numerical difference of the CI-CS value for the treatment period (Visit 2 versus Visit 4) minus the CI-CS value for the washout period (Visit 4 versus Visit 6). This endpoint appropriately captured improvement in symptoms during the treatment period together with worsening of symptoms during the washout period. A sample size of 30 patients would provide the trial with 76% power, at a one-sided significance level of 5%, to detect a mean effect of at least 0.45 in the primary endpoint (assuming a standard deviation 1.02). The 76% power was a consequence of pragmatic considerations in relation to clinically relevant outcomes on the CI-CS scale following treatment with IB1001 and subsequent washout and was viewed as being acceptable for the purpose of assessing a true treatment-related effect. With respect to the primary and secondary outcome measures, this would provide a sufficient dataset, taking account of the rarity of these neurological conditions, and was viewed as being acceptable for assessing a true treatment-related effect. The primary analysis was performed according to the modified intention-to-treat (mITT) principle, used to estimate the treatment effect regardless of early patient discontinuation and provides a perspective of the treatment effect across the entire population. The mITT analysis set was defined as all patients who receive at least one dose of the study drug and with one video recording during the baseline period (Visit 1 or 2, or both) and treatment period (Visit 3 or Visit 4, or both). The mITT analysis utilized a last observation carried forward approach for the primary CI-CS endpoint which implies that the CI-CS value for Visit 4 to Visit 6 is assigned the value 0 (stable) if both videos from the washout period (Visit 5 and Visit 6) were not available. The analysis of the primary endpoint was based on a single sample one-sided Wilcoxon comparing the mean of the CI-CS differences with zero. The null hypothesis is that the mean is ≤ 0, with the alternative hypothesis that this mean is > 0 and the test will be conducted at the one-sided 5% significance level. Non-parametric 90% confidence intervals were constructed using the Hodges–Lehmann method [14]. Secondary endpoints were evaluated either statistically based on a single sample t-test or a single sample Wilcoxon signed rank test or descriptively, as pre-defined in the statistical analysis plan (SAP). There was no formal hierarchical structure defined for the secondary endpoints and results on these endpoints should therefore be viewed as exploratory. For each of the primary and secondary endpoints, there were separate analyses within key subgroups pre-defined in the SAP. These subgroups are listed in Fig. 2. The safety analysis set (SAF) consisted of all patients who received at least one dose of the study drug. The safety, integrity, and feasibility of the trial were monitored by an independent data safety monitoring board (DSMB) consisting of three independent, non-participating members (including two clinicians and a statistician).

## Results

Between 04 September 2019 and 30 January 2020, thirty-four participants were screened, and thirty-three subjects were enrolled per protocol. The demographic and baseline clinical characteristics of the patients are presented in Table 2.

**Table 2:** Demographics and baseline characteristics

Age (years)			Mean (SD)			28.8 (15.1)
			Median			29
			Q1			14
			IQR			24.5
			Q3			38.5
			Range			7–64
Ethnicity, *n* (%)		Asian			1 (3.0%)	
		White			30 (90.9%)	
		Other			2 (6.1%)	
Gender, *n* (%)	Male				24 (72.7%)	
	Female				9 (27.3%)	
Age Group, *n* (%)	Paediatric (< 18 years)				10 (30.3%)	
	Adult (≥ 18 years)				23 (69.7%)	
Dose, *n* (%)	Age 6–12 years—15 to < 25 kg—2 g/day				1 (3.0%)	
	Age 6–12 years—25 to < 35 kg—3 g/day				2 (6.1%)	
	Age 6–12 years → = 35 kg—4 g/day				2 (6.1%)	
	Age ≥ 13 years—4 g/day				28 (84.8%)	
Geographic Location, *n* (%)	USA				2 (6.1%)	
	Europe				31 (93.9%)	
Miglustat at baseline, *n* (%)		Yes			30 (90.9%)	
		No			3 (9.1%)	
Selected primary anchor test, *n* (%)	8 Meter Walk Test (8MWT)			12 (36.4%)		
	9 Hole Peg Test-Dominant Hand (9HPT-D)			21 (63.6%)		

Thirty-two patients qualified for the primary mITT analysis set (96.9%), which included all patients dosed who had at least one treatment visit (Visit 3 or Visit 4). One patient was withdrawn after Visit 2 due to an unrelated adverse event (epileptic seizures, for which the patient needed to commence a medication the PI believed could confound the safety and efficacy analysis of the trial). One patient was withdrawn between Visit 3 and Visit 4 following an unrelated serious adverse event (fall, resulting in head injury/trauma and two broken ribs). The median duration of NALL treatment was 43 days (range: 35–133), with a mean duration of exposure of 51.7 days. The COVID-19 pandemic impacted the duration of treatment/post-treatment washout for select patients, and patients were dosed/on washout until it was safe and feasible to conduct an on-site end of treatment/washout visit in adherence with all COVID-19 local regulations. These deviations due to COVID-19 are reflected by the limited size of the per-protocol population (*n* = 18). Thirty-one patients (93.9%) completed the Parent Study (Visit 6) (last patient last visit occurred 05 August 2020).

**Fig. 3:**
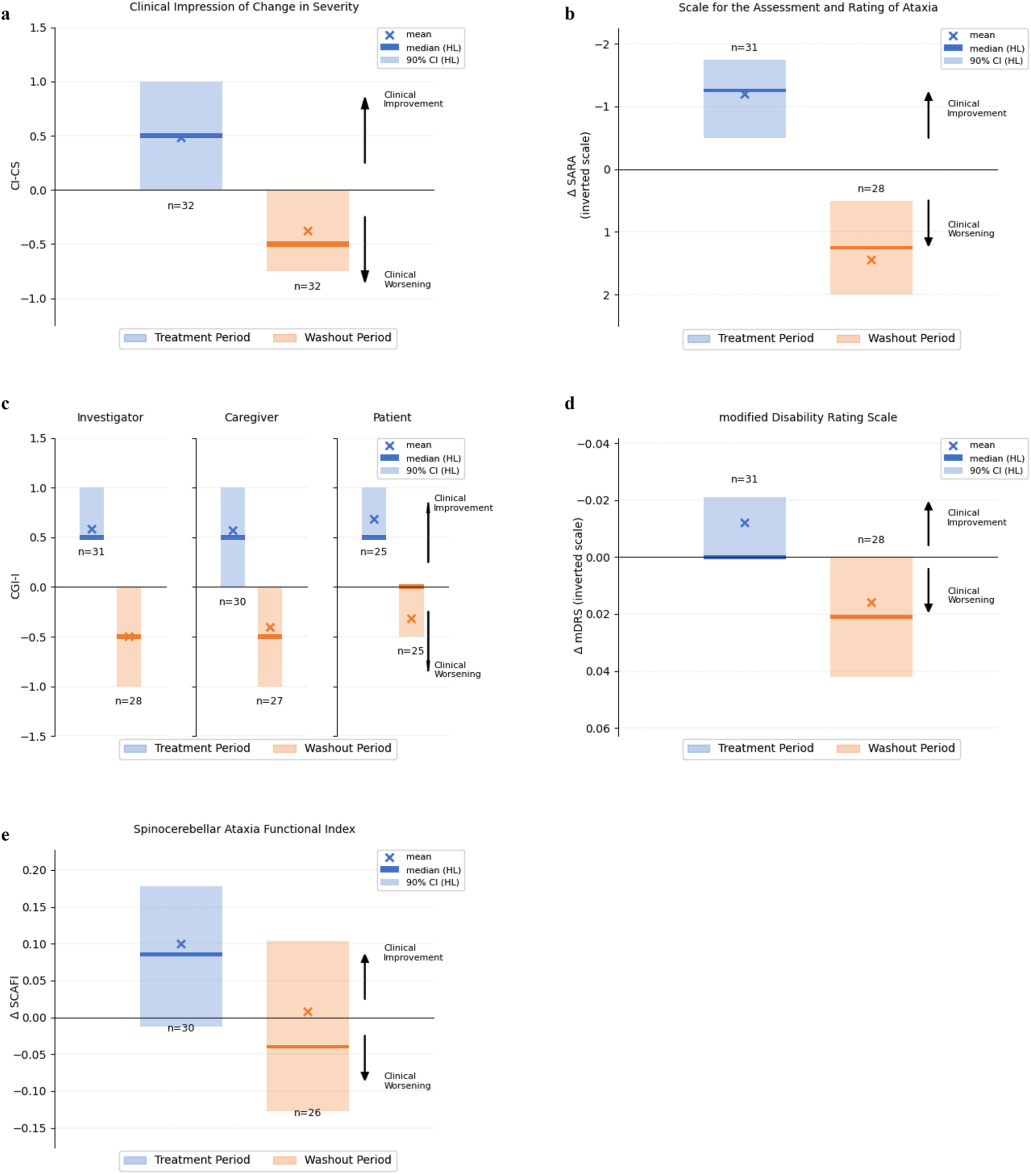
Results of the primary and key secondary endpoints. All analysis based on the mITT population. For each figure, the left-hand column (blue) illustrates CI-CS results comparing baseline to the end of the treatment period; the right-hand column (orange) illustrates the CI-CS results comparing the end of the treatment period to the end of the washout period. The vertical extent of the column represents the 90% Hodges–Lehman (HL) confidence interval of the CI-CS; a solid line is used to indicate the Hodges–Lehman median estimator, and a cross symbol indicates the Mean response. **A** Results of the primary endpoint: Clinical Impression of Change in Severity (CI-CS), based on a 7-point scale, ranging from − 3, “significantly worse”, 0, “no change”, to + 3 to “significantly improved”. **B** Results on the secondary endpoint: Scale for the Assessment and Rating of Ataxia (SARA); **C** results on the secondary endpoint: Clinical Global Impression of Change—Physician, Caregiver, Patient; based on a 7-point scale, ranging from − 3, “significantly worse”, 0, “no change”, to + 3 to “significantly improved”; **D** results on the secondary endpoint: Modified Disability Rating Scale (mDRS); **E** results on the secondary endpoint: Spinocerebellar Ataxia Functional Index (SCAFI)

The CI-CS primary endpoint of the study reached statistical significance with *p* = 0.029 with mean value = 0.86 (SD = 2.52, median = 1.0) and Hodges–Lehmann 90% confidence interval (CI) (0.25, 1.75). There were no missing values for CI-CS for the treatment period although there were four missing values for CI-CS during the washout period (Fig. 3A).

The CI-CS component for the treatment period (Visit 4 versus Visit 2) had a mean value of 0.48 (SD = 1.34 median = 1.0), showing on average an improvement in the patients’ condition over that period, while the CI-CS component for the washout period (Visit 6 versus Visit 4) had a mean value of -0.38 (SD = 1.45, median = − 0.25) showing on average a worsening. There was no difference observed between the CI-CS comparing the baseline and washout visits (Visit 6 versus Visit 2): mean value 0.063 (SD = 1.32, median = 0, *n* = 30). This reinforced the treatment effect, but also demonstrated the absence of a learning effect on the CI-CS anchor tests. The CI-CS was demonstrated to be a reliable instrument, with high inter-rater consistency (0.80 correlation).

A Forest plot (Fig. 2) is included to display results for the primary endpoint in the pre-defined subgroups showing Hodges–Lehman median estimates and corresponding 90% confidence intervals calculated where possible. There is some variation as would be expected given the small sample sizes but there is no evidence that would suggest a differential treatment benefit across the population as a whole. Instead, across all subgroups, there was a consistent trend of improvement during treatment and deterioration during the washout.

The mean total SARA score at baseline (Visit 2) was 14.47 (SD = 7.14, median = 12.74, *n* = 32) and encompassed a full range of disease-severity (min = 5, max = 33). The mean change in SARA score during the treatment period (Visit 2 to Visit 4) was − 1.19 (SD = 2.02, median = − 1.00, *n* = 31) with 90% CI (− 1.75, − 0.50) *p* = 0.001, demonstrating an improvement on cerebellar signs and neurological symptoms. The mean change in SARA score during the washout period (Visit 4 to Visit 6) was 1.45 (SD = 2.56, median 0.75, *n* = 28) with 90% CI (0.50, 2.00), *p* = 0.002, showing a deterioration (Fig. 3B). There was no difference observed between the baseline visit to the washout visit (Visit 2 to Visit 6); the mean change in score was 0.25 (SD = 2.60, median = 0, *n* = 28) with 90% CI (1.1, − 0.59) *p* = 0.308. This further reinforced the treatment effect, but also demonstrated the absence of a learning effect on the SARA subdomains.

The CGI-Change is presented around a reference value of 0 = no change, so that for example − 1 = minimally worse and + 1 = minimally improved. The investigator, caregiver, and patient CGI-C were consistent and showed an average improvement during the treatment period and deterioration during the washout period (Fig. 3C). For the investigator’s CGI-C, the mean change from Visit 2 to Visit 4 was 0.6 (SD = 0.9, median = 0, *n* = 31) with 90% CI (0.5, 1.0) *p* < 0.001 while the mean change from Visit 4 to Visit 6 was − 0.5 (SD = 0.9, median = 0, *n* = 28) with 90% CI (− 1.0, 0) *p* = 0.006. For the caregiver’s CGI-C, the mean change from Visit 2 to Visit 4 was 0.6 (SD = 1.0, median = 0, *n* = 30) with 90% CI (1.0, 0.0) *p* = 0.005, while the mean change from Visit 4 to Visit 6 was − 0.4 (SD = 1.1, median = 0, *n* = 27) with 90% CI − 1.0, 0.0) *p* = 0.038. For the patient’s CGI-C, the mean change from Visit 2 to Visit 4 was 0.7 (SD = 1.0, median = 0.5, *n* = 25) with 90% CI (0.5, 1.0) *p* = 0.003, while the mean change from Visit 4 to Visit 6 was − 0.4 (SD = 0.9, median = 0, *n* = 25) with 90% CI (− 0.5, 0.0) *p* = 0.034.

The mean total SCAFI score at baseline (Visit 2) was − 0.3011 (SD = 1.0405, median = − 0.1362, *n* = 32). The score during the treatment period showed a trend towards improvement, with a mean change (Visit 2 to Visit 4) of 0.0995 (SD = 0.3058, median = 0.0890, *n* = 30) with 90% CI (− 0.0123, 0.1777) *p* = 0.084. The mean change in score during the washout period (Visit 4 to Visit 6) was 0.0076 (SD = 0.3584, median − 0.0621, *n* = 26) with 90% CI (− 0.1269, 0.1031) *p* = 0.315. There was a trend for improvement on the 8MWT (mean − 1.093, 90% CI − 1.53, 0.05, *p* = 0.065) and PATA test (mean 0.95, 90% CI 0.0, 1.75, *p* = 0.076) on medication. As reported in other neurological disorders, the quantifiable time-based SCAFI assessments may not best capture clinically meaningful changes in functioning or quality of life; hence, the CI-CS assessment was developed [15].

The mean mDRS at baseline was 0.467 (SD = 0.155, median = 0.458, *n* = 32). Change from baseline through to Visit 4 showed a small improvement in terms of disability on average with a mean change of − 0.012 (SD = 0.050, median = 0, *n* = 31), 90% CI (− 0.021, 0.0) *p* = 0.121 with a mean change from Visit 4 to Visit 6 (washout) of 0.016 (SD = − 0.051, median = 0, *n* = 28), 90% CI (0.0, 0.042), *p* = 0.056 on average showing a slight worsening in terms of disability.

A treatment-emergent adverse event (TEAE) was any adverse event (AE) that appeared or worsened after study treatment began (i.e. in the treatment or washout period). The distribution of TEAEs is shown in Supplementary Table 2**.** There were no AEs with an incidence > 10%. Seven related AEs were reported for 4 patients, including: flatulence, diarrhoea, increased feeling of hunger, rash (twice for 1 patient), and aggressive behaviour accompanied by restlessness. The events were transient and manageable. No serious adverse reactions were reported. No deaths occurred during the study. Results of plasma and urine tests, vital signs, and ECG recordings were normal or rated as clinically non-significant. Adherence was high as shown by treatment compliance and the regular urine analyses for prohibited medication.

## Discussion

Here we report the results of a Phase II clinical trial investigating the safety and efficacy of the modified amino acid NALL for NPC disease in patients aged 6 years or older. The major findings of the IB1001-201 trial are: first, NALL improved symptoms, including gait and stance, upper extremity function, and fine motor skills, which worsened during the post-treatment washout. Second, consistent with its pharmacological action, NALL improved cerebellar signs and functioning after 6 weeks. Third, improvement of neurological status was observed across all demographics of patients (age, gender, age of onset, disease severity, etc.) establishing the rationale for NALL to be used as a treatment for all NPC patients. Fourth, the low frequency (7 related AEs in 4 patients of 32 participants) and the transient, mild nature of these AEs inform a favorable benefit-risk profile.

Methodologically, to elaborate a novel clinical endpoint, the CI-CS based on videos of the 8MWT or the 9HPT-D was used which was rated (0 ± 3) by blinded, centralized neurologists. In addition to reducing detection and performance bias for the primary endpoint, the blinded CI-CS served as a metric of clinical importance that could not be obtained from the traditional timed assessments. Instead, raters evaluated clinically meaningful changes in patient’s neurological manifestations which correlate to their level of functioning and quality of life, such as the accuracy and fluidity of movements, spasticity, ataxia, and dystonia for the 9HPT-D, and changes in gait patterns such as balance and postural stability, variability, asymmetry, ataxia, and support for the 8MWT [7].

Of note, 93.8% of patients were on the standard of care agent Miglustat. The administration of NALL therefore showed significant effects beyond Miglustat for the signs and symptoms of NPC. This is in line with the previous observational studies that indicated the additive effect of NALL with Miglustat [3, 4], and studies demonstrating synergistic effects of NALL and Miglustat [5].

NALL’s positive treatment effects directly correlate to its pharmacological action. Animal studies in an *Npc1*^−/−^ mouse model show NALL significantly reduces ataxia when treatment is initiated either symptomatically (from 8 weeks of age) or pre-symptomatically (from 3 weeks of age) [5]. These in vivo studies further show NALL can restore neuronal function and protect against/delay disease progression in multiple neurological brain circuits. Altered glucose and antioxidant metabolism and reduction of cerebellar inflammation have been implicated as potential mechanisms of action [5, 16]. Similar neuroprotective, disease-modifying effects of acetyl-leucine were found in an analogous animal model of another lysosomal storage disease, the Sandhoff mouse [17]. Notably, the dosage used in these in vivo studies (0.1 g/kg/day) is roughly equivalent to the dose used in previous observational clinical studies with the racemate and the IB1001-201 clinical trial.

The clinical manifestations of this, as demonstrated in the IB1001-201 clinical trial, were visualized and captured in terms of improvements or stabilization in very different processes such as ambulation, fine motor skills, speech, and cognition. This is also consistent with previous observational studies with *N*-acetyl-leucine where treatment of 4–6 weeks significantly improved symptoms, functioning, and quality of life in 12 NPC patients, and where long-term treatment of at least 1-year lead to a significant reduction in disease progression and clinical improvement in the majority of patients, indicative of a neuroprotective, disease-modifying effect [3, 4].

The IB1001-201 parent study has several limitations. First, it was not placebo-controlled. As previously reported, the widespread, unlicensed use of the commercially available racemate (*N*-acetyl-dl-leucine; Tanganil™) and even, patient’s access to chemical grade substitutes of NALL, within the NPC is well-known. This severely limited the feasibility of including a placebo-control, given patients and families reluctance to participate in a study where they would be required to washout from this unlicensed medication and receive an inactive treatment for even 50% of the time [7]. To ensure the study remained well-controlled and minimized bias, controls were put in place for the study—including the centralized, blinded review paradigm and use of intra-patient internal control—to enable a complete assessment of the scientific integrity and validity of the results. To minimize potential patient expectation or investigator bias, all aspects of the administration and video recording of the CI-CS anchor tests were standardized to ensure the quality of videos assessed by the blinded raters. Before any patient visit, site personnel were trained on these detailed protocols, including precise verbal instructions, encouragement, break times between test trials, and instructions on which trial to record. Given that the majority of NPC patients enrolled in the IB1001-201 clinical trial featured severe physical impairments with regard to both their fine motor skills as well as balance and gait, and mild to significant levels of cognitive impairment, the potential for a placebo-effect which significantly altered neurological signs and symptoms–the basis of the CI-CS assessment—was considered minimal, ensuring the interpretability of the blinded-raters’ CI-CS assessments.

Second, the novel CI-CS primary endpoint has not been previously used or yet validated. It was implemented, however, due to the methodological limitations of applying pre-existing ataxia scales in heterogeneous diseases, in which cases, the scales may be too broad and therefore not sensitive to capture small but meaningful functional changes [7, 18]. The validated NPC Clinical Severity Scale is only validated to measure disease progression after a minimum of 1 year; thus, it is not sensitive enough to capture or measure change after 6-weeks [19]. Accordingly, the CI-CS was developed to be a more clinically relevant endpoint capable of detecting clinically meaningful treatment effects. All aspects of the CI-CS were standardized and well-defined, and the CI-CS was demonstrated to be a reliable instrument, with high inter-rater consistency.

Third, this study was limited to an investigation of the symptomatic effect of NALL treatment; data from the ongoing Extension Phase will provide further insights into the impact on disease progression and long-term safety. Given that the heterogeneous symptoms of NPC are serious, debilitating, and significantly impact quality of life, managing the neurological symptoms of NPC represents an important and meaningful treatment paradigm for patients.

In conclusion, this study provides strong support for the use of NALL as a symptomatic treatment for NPC. Consistent with previous non-clinical and observational clinical studies, NALL demonstrated a significant and clinically meaningful improvement of neurological symptoms, functioning, and quality of life in pediatric and adult NPC patients and was safe and well-tolerated, contributing to a favorable benefit-risk profile for the treatment of this serious, debilitating disease. Given NPC is a progressive, life-threatening condition with limited or no approved medicinal treatments, and no symptomatic treatments, and considering the totality of the evidence available for NALL, there is an urgency for treatment to be available for patients before the window of therapeutic opportunity is lost.

## Supplementary Information

Below is the link to the electronic supplementary material.

Supplementary file1 (DOCX 18 kb)

## Data Availability

All authors had full access to all the data in the study and had final responsibility for the decision to submit for publication. The study documents related to the study, and datasets generated and analyzed during the current study are not publicly available. No individual, de-identified participant data will be shared.
