# Supplementary material for: Efficacy and safety of N-acetyl-l-leucine in Niemann–Pick disease type C
Source: J Neurol. 2021 Aug 13;269(3):1651–62. doi: 10.1007/s00415-021-10717-0 (PMC8361244; doi:10.1007/s00415-021-10717-0)
Supplement: Supplementary file 1 — Supplementary file1 (DOCX 18 kb) [file 415_2021_10717_MOESM1_ESM.docx]

|  | **Video A** | **Video B** | **Video C** |
| --- | --- | --- | --- |
| **Order 1** | Visit 2 | Visit 4 | Visit 6 |
| **Order 2** | Visit 2 | Visit 6 | Visit 4 |
| **Order 3** | Visit 4 | Visit 2 | Visit 6 |
| **Order 4** | Visit 4 | Visit 6 | Visit 2 |
| **Order 5** | Visit 6 | Visit 2 | Visit 4 |
| **Order 6** | Visit 6 | Visit 4 | Visit 2 |

**Supplementary Table 1 –Random Video Analysis Order Sequences (Visit 1-6) for videos.** For each patient, the raters assessed the CI-CS for three pairs of videos: the change from Visit 2 to 4 (baseline to end treatment), Visit 4 to 6 (end of treatment to end of washout), and Visit 6 to Visit 2 (end of treatment to baseline). For each patient, MCL generated a random number (1-6) via [*RANDOM.ORG*](http://www.random.org/) which corresponded to a video analysis order sequence.

| **System Organ Class** | **Total (N=33)** | |
| --- | --- | --- |
| **Preferred Term** | **n (%)** | **m** |
| Any treatment emergent adverse event | 24 (72.7%) | 58 |
| Infections and infestations | 9 (27.3%) | 10 |
| Gastroenteritis | 2 (6.1%) | 2 |
| Lower respiratory tract infection | 2 (6.1%) | 2 |
| Nasopharyngitis | 1 (3.0%) | 1 |
| Rhinitis | 3 (9.1%) | 3 |
| Upper respiratory tract infection | 2 (6.1%) | 2 |
| Nervous system disorders | 8 (24.2%) | 10 |
| Balance disorder | 1 (3.0%) | 1 |
| Coordination abnormal | 1 (3.0%) | 1 |
| Dementia | 1 (3.0%) | 1 |
| Drooling | 1 (3.0%) | 1 |
| Dropped head syndrome | 1 (3.0%) | 1 |
| Headache | 1 (3.0%) | 1 |
| Petit mal epilepsy | 1 (3.0%) | 1 |
| Seizure | 3 (9.1%) | 3 |
| Injury, poisoning and procedural complications | 5 (15.2%) | 9 |
| Contusion | 1 (3.0%) | 1 |
| Fall | 3 (9.1%) | 5 |
| Head injury | 1 (3.0%) | 1 |
| Rib fracture | 1 (3.0%) | 1 |
| Traumatic haematoma | 1 (3.0%) | 1 |
| Gastrointestinal disorders | 5 (15.2%) | 8 |
| Anal incontinence | 1 (3.0%) | 1 |
| Diarrhoea | 3 (9.1%) | 5 |
| Dysphagia | 1 (3.0%) | 1 |
| Flatulence | 1 (3.0%) | 1 |
| Respiratory, thoracic and mediastinal disorders | 5 (15.2%) | 5 |
| Cough | 1 (3.0%) | 1 |
| Epistaxis | 2 (6.1%) | 2 |
| Obstructive airways disorder | 1 (3.0%) | 1 |
| Productive cough | 1 (3.0%) | 1 |
| Skin and subcutaneous tissue disorders | 3 (9.1%) | 4 |
| Rash | 2 (6.1%) | 2 |
| Rash pruritic | 2 (6.1%) | 2 |
| Psychiatric disorders | 2 (6.1%) | 3 |
| Aggression | 1 (3.0%) | 1 |
| Restlessness | 1 (3.0%) | 1 |
| Sleep disorder | 1 (3.0%) | 1 |
| General disorders and administration site conditions | 2 (6.1%) | 2 |
| Asthenia | 1 (3.0%) | 1 |
| Fatigue | 1 (3.0%) | 1 |
| Investigations | 2 (6.1%) | 2 |
| Blood alkaline phosphatase increased | 1 (3.0%) | 1 |
| Blood pressure increased | 1 (3.0%) | 1 |
| Metabolism and nutrition disorders | 2 (6.1%) | 2 |
| Dehydration | 1 (3.0%) | 1 |
| Increased appetite | 1 (3.0%) | 1 |
| Musculoskeletal and connective tissue disorders | 2 (6.1%) | 2 |
| Arthralgia | 1 (3.0%) | 1 |
| Neck pain | 1 (3.0%) | 1 |
| Reproductive system and breast disorders | 1 (3.0%) | 1 |
| Ejaculation failure | 1 (3.0%) | 1 |

**Supplementary Table 2 – Distribution of Treatment-emergent adverse events (IB1001-201 - SAF)**
